# Generating highly reflective and conductive metal layers through a light-assisted synthesis and assembling of silver nanoparticles in a polymer matrix

**DOI:** 10.1038/s41598-017-12617-8

**Published:** 2017-09-29

**Authors:** Mohamed Zaier, Loïc Vidal, Samar Hajjar-Garreau, Lavinia Balan

**Affiliations:** CNRS, Institut de Science des Matériaux de Mulhouse, UMR 7361, 15 rue Jean Starcky, 68057 Mulhouse, France

## Abstract

The development of metalized surfaces exhibiting mirror properties and/or electric conductivity without heavy equipments and with low metal charge is a big challenge in view of many industrial applications. We report herein on the photo-assembling of silver nanoparticles (AgNPs) in a polymer matrix, carried out within minutes from an acrylate monomer and silver nitrate at room temperature, under air and without any solvents. The top surface of the material gets converted into a continuous silver thin film and a depthwise concentration gradient of AgNPs is created in the polymer, which images the absorption profile of the actinic UV light in the reactive formulation. This specific assembling of the silver@polymer coating induces excellent reflective and conductive properties. The conductance was observed to strongly increase with increasing the exposure from 3 to 30 min due to the formation of a more and more compact metal film. This coating strategy works with a variety of substrates (textile, paper, glass, wood, plastic and stainless steel). Moreover, on flexible surfaces such as textile, the flexibility was preserved. The possibility to use this kind of nanomaterial as a printing ink, with a much lower metal concentration (3 to 5 wt.%) than concurrent inks, was also demonstrated.

## Introduction

In the past decades, the deposition of metal nanoparticles (MNPs) at the surface of various substrates for applications such as highly reflective coatings or printing techniques has gained wide interest. For example, Ag films can be used as decorative elements and reflector concentrators for solar power generation^1^, as contacts in microelectronics^2^, as antibacterial surfaces^3,4^, and for their reflective and conductive properties^5–8^. Various methods were developed to engineer highly reflective Ag mirrors. The early works of the German chemist Justus von Liebig, based on spraying a glass surface with a solution of Ag^+^ and sugar^9^ is still used in the manufacture of common household mirrors. In 1988, Yogev and Efrima introduced an approach for generating Ag mirrors at a liquid-liquid interface via multilayered metal liquid films (MELLFs) and Ag aggregates were formed at a water/dichloromethane interface^10^. Southward *et al*. developed a thermally cured silver-polyimide films via the *in situ* reduction of silver(I) acetate for a few hours and at 300 °C^11,12^. Electron-beam gun evaporation^13^ can also be used to generate metal mirrors but the method requires severe deposition conditions such as high temperature and high electron gun intensity. Recently, an electrochemically switchable stable and bistable silver mirror was prepared by introducing a thiol-modified indium tin oxide (ITO) electrode in ionic liquids to improve the stabilization of the metallic film^14^. However, the mirror state requires the continuous application of a reductive voltage to avoid the dissolution of the Ag film into the electrolyte.

Another chemical method developed recently to generate Ag mirrors is the fluoride-induced reduction of Ag^+^ cations. It relies on the reduction of a Ag^+^ Lewis acid into Ag(0) by a F^-^, thus generating a Ag mirror^15^. However, this method suffers some drawbacks, e.g. use of only aprotic solvents, additional washing step to remove the excess of reagents for the mirrors, and noteworthy is also that the deposition is feasible only on the surface of reaction containers.

Inkjet printing has also attracted increasing attention due to its potential applications in photovoltaics^16^, light-emitting diodes (LEDs)^17^, sensors^18^, batteries^19^ or smart clothing^20^. Among inkjet printings, the deposition of conductive patterns on substrates like paper, plastic or textile is of high interest for the fabrication of electronic devices such as chemical sensors, field effect transistors (FETs), electrical circuits or for radio frequency identification (RFID)^21,22^. Because the conductivity of materials like polymers, carbon or graphene (10 to 10^2^ S.cm^−1^) is generally lower than that of metals (10^4^ to 10^5^ S.cm^−1^), the use of MNPs, and especially of AgNPs, was recently investigated intensively. The interest in AgNPs is mainly motivated by their unique electric conductive properties, their ease of production compared to AuNPs, high stability and low cost^23,24^. Recent reports demonstrated that AgNPs inks can be prepared using preformed poly(acrylic acid) or poly(vinylpyrrolidinone) while AgNPs obtained by reduction of an Ag^+^ salt using ethylene glycol at high temperature or monoethanolamine as reducing agents or via a modified Tollens’ process require ammonium hydroxide and formic acid^25–29^. However, the need to synthesize AgNPs in advance and/or the use of hazardous chemicals or organic solvents restricts the use of these methods.

During recent years, our research group developed MNPs and metal-polymer nanocomposites (silver, gold or palladium) using a photo-induced approach. Because it allows activation of chemical reactions at ambient temperature, light acts without inducing collateral damages due to heating of the surrounding media. This approach offers the advantage over concurrent thermally activated processes to generate MNPs *in situ* and in a photosensitive formulation or in a polymer matrix. Therefore, it has become highly valuable for elaborating metal-polymer nanocomposites containing homogenously dispersed MNPs^30–32^.

The self-assembly of MNPs has also recently emerged as a promising way of generating tunable optical or plasmoniques devices^33,34^. However, the perfect control of the spacial distribution of MNPs and their assembling is clearly a challenge for the synthesis of 3-dimensionally (3D) shaped nanomaterials.

In this paper, we report on an efficient, *in situ*, one-step and all-photoinduced approach to produce metal mirrors and conductive coatings at room temperature and under air. This is conducted by spatially controlling and assembling MNPs in a 3D polymer network. Indeed, the depthwise arrangement of AgNPs, i.e. tuning their density from the surface to the depths of the coating, is obtained through a kinetic key, which is activated by the absorption of the actinic light. In this way and under particular experimental conditions, it is possible to generate a continuous thin metal film at the coating top surface exhibiting excellent electric conductivity and light reflectivity i.e. a mirror with a sub-wavelength flatness. To the best of our knowledge, there is no current research and development on such advanced coatings relying on an *in situ* generation of metal nanoparticles and simultaneous crosslink of a polymer matrix through a photo-assisted process. And on top of that, this approach offers the possibility to control the in-depth distribution of the particles in the polymer layer.

## Results and Discussion

A one-step photo-induced approach was developed to synthesize silver@polymer nanoassemblies on a variety of substrates (see Figure S1). It requires only a photosensitive formulation and a UV source. The photosensitive formulation - metal precursor Ag^+^ (5 wt.%), photosensitizer (0.5 wt.%) and acrylate monomer - was leaved for 60 min at ambient temperature under magnetic stirring. This step ensured total dissolution of the metal precursor and photosensitive generator of free radicals. The highly reactive free radicals stemming from homolytic cleavage of the photosensitive precursor are used to reduce silver cations to AgNPs. In parallel, these species initiate the photopolymerization of acrylate units, thus generating the host polymer network^35–39^. Controlling the kinetics of these two concurrent processes, *in situ* photo-reduction and photo-polymerization, is the key issue of the synthesis of silver@polymer nanoassemblies.

Two configurations were used to obtain the nanomaterial: (i) direct illumination of the sample (sensitive formulation) in the open air, (ii) lamination of the sample between two glass slides, then illumination for a few seconds (5 sec., unless otherwise stated) and finally delamination by removing the cover slide and continuation of the illumination in the open air. This latter strategy was used to shield the radicals from being quenched by oxygen and to evaluate its detrimental effect onto the assembling process of silver particles during the initial stages of the reaction before the formulation gelled.

**Figure 1:**
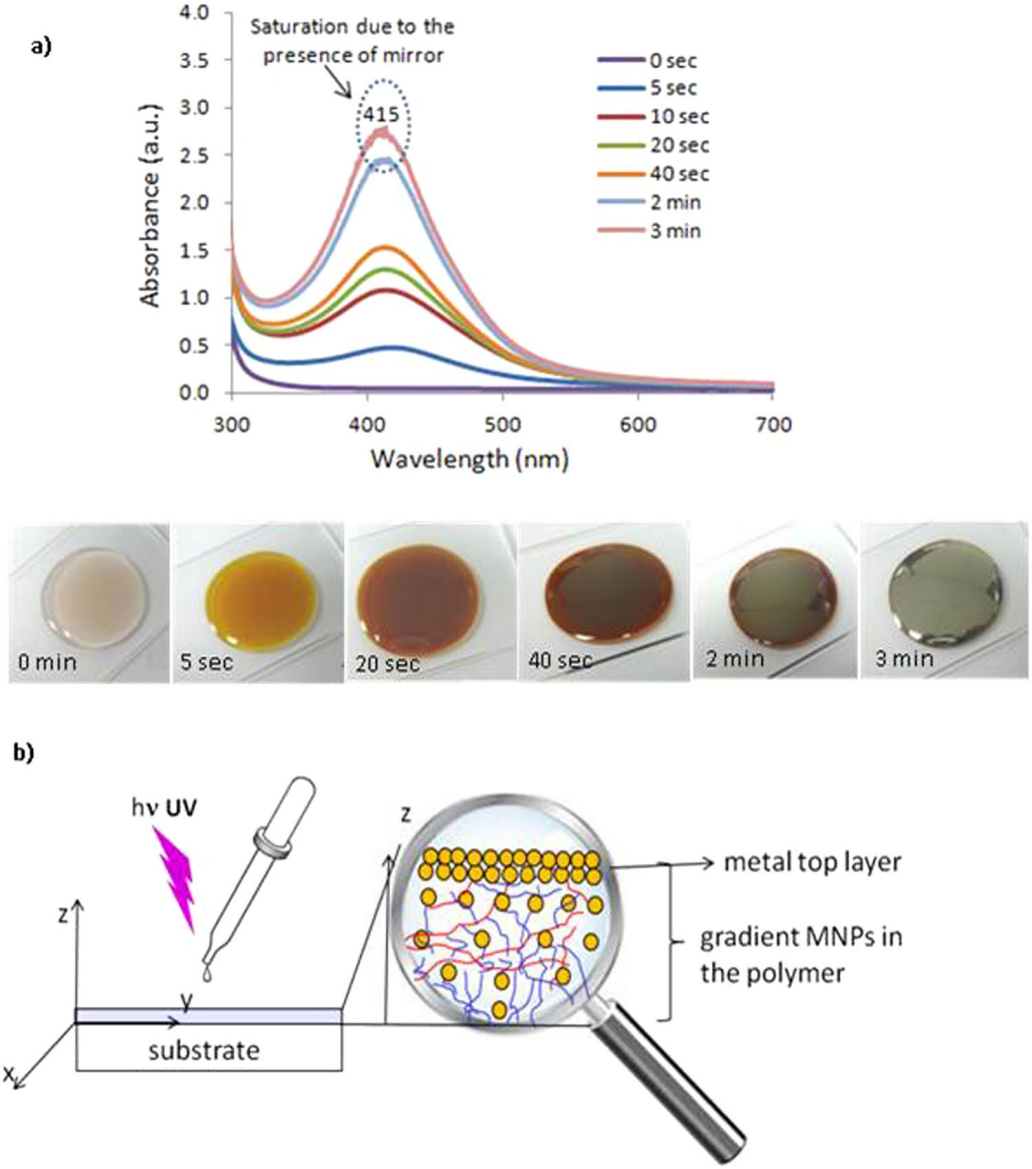
(**a**) Absorption spectra of the silver nanoassembly in the polymer matrix before (0 sec) and after increasing light exposures (5 sec, 10 sec, 20 sec, 40 sec, 2 min and 3 min) and photographic images of the corresponding samples. (**b**) Scheme describing the formation of the MNPs nanoassembly.

Figure 1a shows the UV-Vis spectra obtained before (0 sec) and after light exposure (until 3 min exposure) and the photographic images of the corresponding samples. In the absorption spectrum at 0 sec, no absorption band is visible in the visible range. The synthesis of silver nanoparticles starts a few seconds (less than 5 sec) after exposing the photosensitive formulation to UV light and under air. At the same time, the characteristic absorption associated to the surface plasmon resonance of AgNPs developed. The intensity of this peak centered at 415 nm, increases as the illumination proceeds, up to 3 min-exposure. From this moment, the process saturated due to the generation of a totally reflecting layer at the top surface of the coating (see Fig. 1a spectra and images). The photographic images show the characteristic color of the small-sized AgNPs after 5 sec-exposure and its intensification as the photoreduction proceeds.

At the beginning of the irradiation, light penetrates in all the depth of the photosensitive formulation. As the irradiation proceeds, AgNPs resulting from the photo-reduction of metal cations are generated (Fig. 1a). Their in-depth distribution is not homogeneous due to the attenuation of the actinic light propagating from the surface to the bottom of the layer (due to Beer-Lambert attenuation). Moreover, as the photoreduction proceeds, NPs contribute to an extra internal filter effect due to their own absorption (plasmon resonance). The conjunction of these two effects, which affect both the intensity and penetration depth of the actinic light in the formulation, is at the root of the in-depth distribution shape of NPs in the metal-polymer layer (see Fig. 1b).

As irradiation proceeds, the gradient intensifies, to such an extent that it progressively confines the reduction reaction to the close vicinity of the top surface of the coating. That way, the concentration of NPs in this region becomes high enough to allow aggregation of the nanoparticles. Thus, after a 2 min-exposure, the top surface of the coating turns more and more reflective (see Fig. 1). If the UV exposure is kept on, the concentration in NPs grows up and they ends in coalescing until a continuous reflective silver layer is obtained at the top surface of the coating (see Fig. 1).

Clearly, the key issue of this nanoassembling process is the kinetic coupling between the formation of NPs and that of the photo-crosslinking of the polymerizable matrix. This latter has to proceed rapidly enough to allow stabilization of the synthesized nanoparticles but not too fast to impede the formation and the organization of those to come. Moreover, this kinetic coupling evolves over time and in depth due to the feedback associated to the internal filter effect of AgNPs. As a result of this specific assembling process in the metal-polymer nanocomposite material, an in-depth structuration of the coating develops (Fig. 1b). Thus, a veritable reflective metal layer forms on the top of the coating (Fig. 1). Quite interestingly, when the substrate is transparent to the actinic wavelength, the same in-depth structuration can also be generated on the reverse direction (at the bottom interface), by irradiating the layer upside down (see Figure S2).

**Figure 2:**
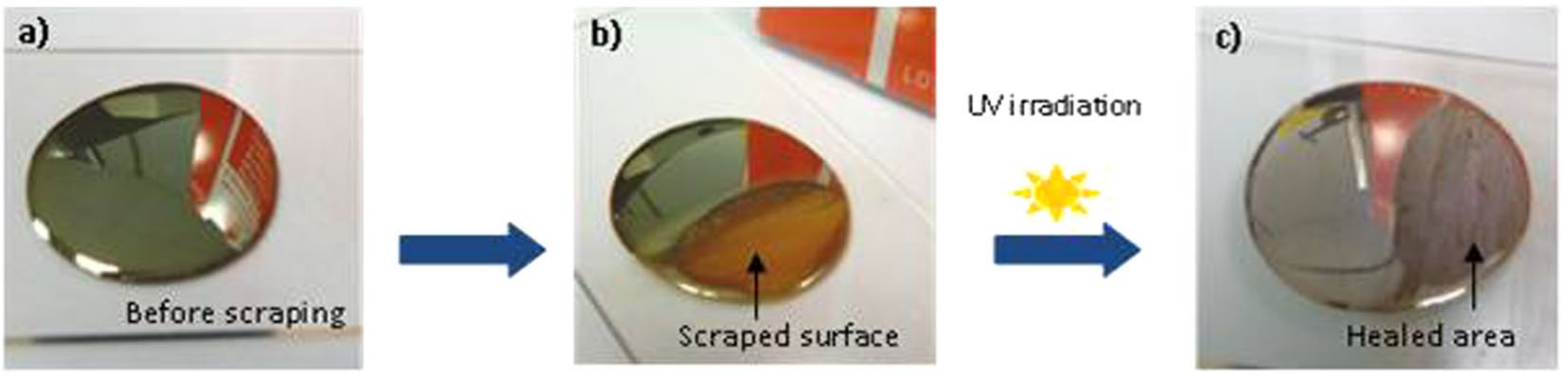
Photographic images of the corresponding samples: (**a)** before and (**b)** after scraping and (**c)** after self-healing of the metal layer.

Another very attractive feature of this material is its ability to “self-heal” defects resulting from cracking or accidental peeling and the consequence of which is an irreversible loss of the properties i.e. reflectivity and electric conductivity. Thus, if an as-prepared metal layer (Fig. 2a) was scratched with a steel punch or emery cloth so as to simulate a deterioration (Fig. 2b), irradiating the damaged area under the conditions used to generate the original layer allows the material to heal (Fig. 2c). Indeed, it can be seen that after scraping, the surface is yellowish-brown and after UV exposure, the typical metallic sheen appears again, the extra silver fills up with the groove and the metal surface is reconstructed. This was confirmed also by TEM analysis (data not shown in the paper). Depending on the circumstances, the optical quality of the healed area may not be perfect with a downgrade mirroring effect, but the electrical conductivity itself, is completely restored. The question of the origin of silver cations involved in this healing process will be dealt with further down in the document.

The metal@polymer nanoassemblies were characterized by XRD to confirm the formation of Ag(0) (Figure S3). XRD patterns were recorded on samples obtained with 3- and 30- min UV exposure in open air. The spectra show all the diffraction peaks indexed to a cubic crystalline structure of Ag(0) and assigned to (111), (200), 220 and (311) planes^40^ with a 2θ values of 38°, 44°, 64° and 77°, respectively (see Figure S3). The broad peak around 21° corresponds to the polymer matrix^41^. It is worth of notice that, under the experimental conditions prevailing, increasing the UV exposure results in an increase of the concentration of Ag(0) nanoparticles but does not affect their crystalline structure (Figure S3).

**Figure 3:**
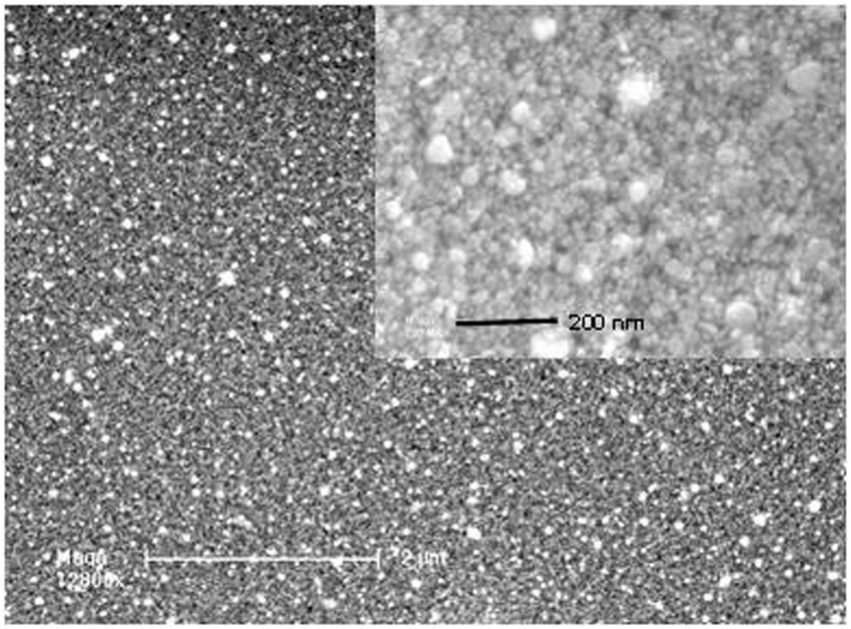
SEM image taken at the surface of the silver mirror and magnification in the insert.

Figure 3 shows the images recorded by SEM on the surface of the nanomaterial as it is. The image shows a good dispersion of AgNPs obtained on the surface of the sample (Fig. 3) with a diameter ranging from 10 to 70 nm. Chemical analysis by energy-dispersive X-ray spectroscopy (EDXS) confirms the presence of signals corresponding to silver, carbon and oxygen; the last two ones are mainly related to the polymer matrix (see Figure S4).

TEM micrographs were obtained after microtome cutting of the samples on the cross section of the layers. The influence of several experimental parameters (composition of the reactive formulation, exposure time, under lamination or totally under air) was investigated.

**Figure 4:**
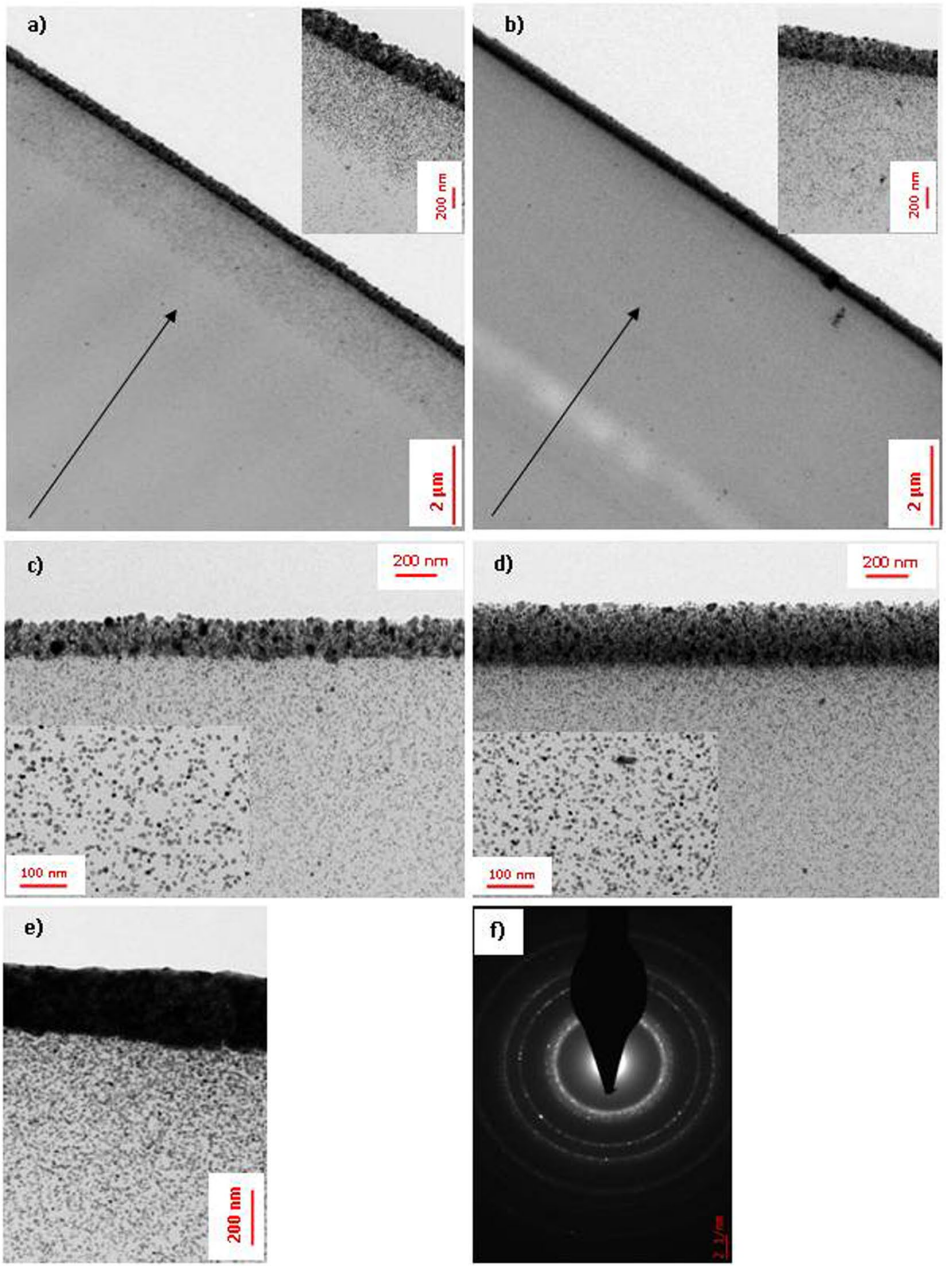
TEM micrographs of cross-sections of the silver nano-assemblies: (**a)** open to the air and (**b)** 5 sec laminated and then open to the air, 5 wt.% Ag^+^ and 3 min exposure (insert: magnification of the top of the samples); (**c)** 3 wt.% and (**d)** 5 wt.% Ag^+^, 3 min UV exposure (insert: magnification of the core of the samples); (**e)** 5 wt.% Ag^+^ with increased UV exposure time (30 min); (**f)** SAED patterns of the top layer.

The influence of oxygen from the atmosphere was firstly studied. For this, two series of samples were compared, one with an open to the atmosphere configuration and the other, laminated for the 5 first seconds of illumination and then open to the air. The TEM analysis (Fig. 4a and b) confirms in both configurations, the spatial organization of the AgNPs i.e. a gradient NPs density from the surface to the depth of the coating. Moreover, when the illumination was carried out in the open air configuration from beginning to end, the gradient is more pronounced with three distinct areas i.e. from the surface to the core of the composite coating, a continuous layer made of coalescing AgNPs (a few hundred nanometers), an intermediate composite later made of silver NPs homogeneously dispersed in the crosslinked polymer structure (typ. 1–2 micrometers) and a background polymer layer containing a small amount of silver NPs (see Fig. 4a).

The TEM micrographs of Fig. 4c and d correspond to silver@polymer samples produced with formulations containing 3 and 5 wt.% Ag^+^, to evaluate the influence of the metal precursor concentration. The illumination was carried out in the laminate-then-open configuration. As can be seen in both of them, the nanoparticles are very well dispersed and do not show aggregation in the depth of the layer and they are quite monodisperse in size with a diameter of ca. 7 ± 0.6 nm. However, the thickness of the metal top layer increases from 170 nm to 240 nm with increasing Ag^+^ concentration (see Fig. 4c and d).

Figure 4e shows a sample obtained with the same configuration as that of Fig. 4d except the fact that the irradiation was carried on for 30 minutes. Comparing Fig. 4d and e, reveals that the irradiation time is a very important factor of the assembling process. Increasing it from 3 min to 30 min has an influential impact on the in-depth organization of the nanoparticles and more specifically, on the top layer where the particle density is high enough to form a dense metal layer in which the metal particles are in close contact. With the 30 min-exposure, the top metal layer is ca. 250 nm thick; as can be seen, all of the silver nanoparticles of the underlying layer are spherical, well dispersed and homogeneous in size (10–11 nm) (Fig. 4e).

The SAED patterns, on the top of the sample, confirm the presence of Ag(0) with a face centered cubic (fcc) structure. Figure 4f shows the rings indexed to the (111), (200), (220), (311) and (331) crystalline planes of Ag(0).

XPS analysis was carried out to study the chemistry of the silver@polymer nanoassembly. Two measurements were performed, the first one on the surface and the second one in depth of the coating in order to evaluate the change between surface and volume. The surface of the sample was scraped with a microtome in order to identify the chemical composition of the sample in the depth of the film. The XPS spectra obtained both on the surface and in the depth of the sample exposed 3 minutes to UV light (see Figure S5) confirm the results obtained by EDXS analysis i.e. only Ag, C and O are detected, the last two ones being assigned to the polymer matrix. The Ag 3d5/2 spectrum (368.31 eV) is reported on the Figure S5c clearly reveals the presence of Ag(0) at the surface, but also a slightly oxidation of the silver nanoparticles to AgO (368.50 eV). It is worth of notice that the distinction between metal Ag(0) and other silver chemical states such as AgO, was made from the calculations of the Auger parameters Ag M_4_N_45_N_45_^42^. In this regard, it is important to mention that the XPS analysis was carried out few weeks after the synthesis and the sample was kept in the open air with no special precaution. The presence of the same three elements (Ag, C, O) was confirmed also in the heart of the coating, and the Ag 3d5/2 spectrum shows only the peak corresponding to Ag(0) (368.06 eV). One can then conclude that the nanoparticles trapped in the matrix are sheltered from oxidizing due to the cross-linked structure of the polymer. The XPS analysis confirmed also that the abundance of Ag(0) in the top layer (22.68%) exceeds by far that in the depth of the nanomaterial (1.29%) which also confirms the gradient organization of the Ag NPs. Since no other forms of silver are visible, the reduction of Ag^+^ can be postulated to be complete. Table 1, shows the position of the Ag 3d5/2 peaks and the atomic concentrations of metal and oxidized silver.

**Table 1:** Ag 3d XPS fitting and the relative atomic concentrations.

Sample	Block Id	Name	Binding energy (eV)	Peak area	Percent at. %
Surface	Ag3d	Ag3d5/2	368.31	25.46	22.68
	Ag3d	Ag3d5/2 AgO	368.50	6.03	5.38
In depth	Ag3d	Ag3d5/2	368.06	1.31	1.29

The XPS analysis allows the origin of the Ag^+^ ions used to self-heal the metal top layer to be identified; indeed, they come from the slight oxidation of Ag(0) by the air at the surface of the coating material. This self-healing process could be of the utmost importance for high added value applications.

With a view to evaluating the photopolymerization of the PEGDA acrylate monomers and also the influence of silver on the curing kinetics of PEGDA, RT-FTIR measurements were carried out both in the presence and the absence of Ag^+^ (film thickness: 24 µm, in the open air and exposed to a Hg–Xe source, fluence: 20 mW/cm^2^) (Figure S6). The degree of conversion of the acrylate functions was estimated by monitoring the disappearance of the characteristic double bond of the reactive group at 1660 cm^−1^. Upon irradiation, an induction period was first observed, more pronounced in the silver free sample. It corresponds to the well known quenching of the photoinitiator in its excited triplet state by molecular oxygen. After total consumption of dissolved oxygen, the conversion rate undergoes a clear acceleration and reaches 86% in the presence of silver and 71% without silver, after 150 sec irradiation. It can be seen that addition of Ag^+^ (3 and 5 wt%) into the liquid photopolymerizable formulation increases both the rate of polymerization and also the ultimate degree of conversion of the monomer units.

The acceleration of the polymerization kinetics is probably related to the fact that the silver seeds stemming from the nucleation process are readily oxidable by molecular oxygen. Due to this process, the stationary concentration of dissolved oxygen in the formulation is substantially less than in the absence of silver, hence a reduction of the spurious quenching of initiating and chain carrier species and thus, an acceleration of the polymerization kinetics. As regards the improvement of the ultimate conversion degree of the monomer units, it might be due to the non-radiative deexcitation of the plasmon that induces local heating around silver nanoparticles and as a consequence, favors the diffusion of unreacted polymerizable units towards living macroradicals.

**Figure 5:**
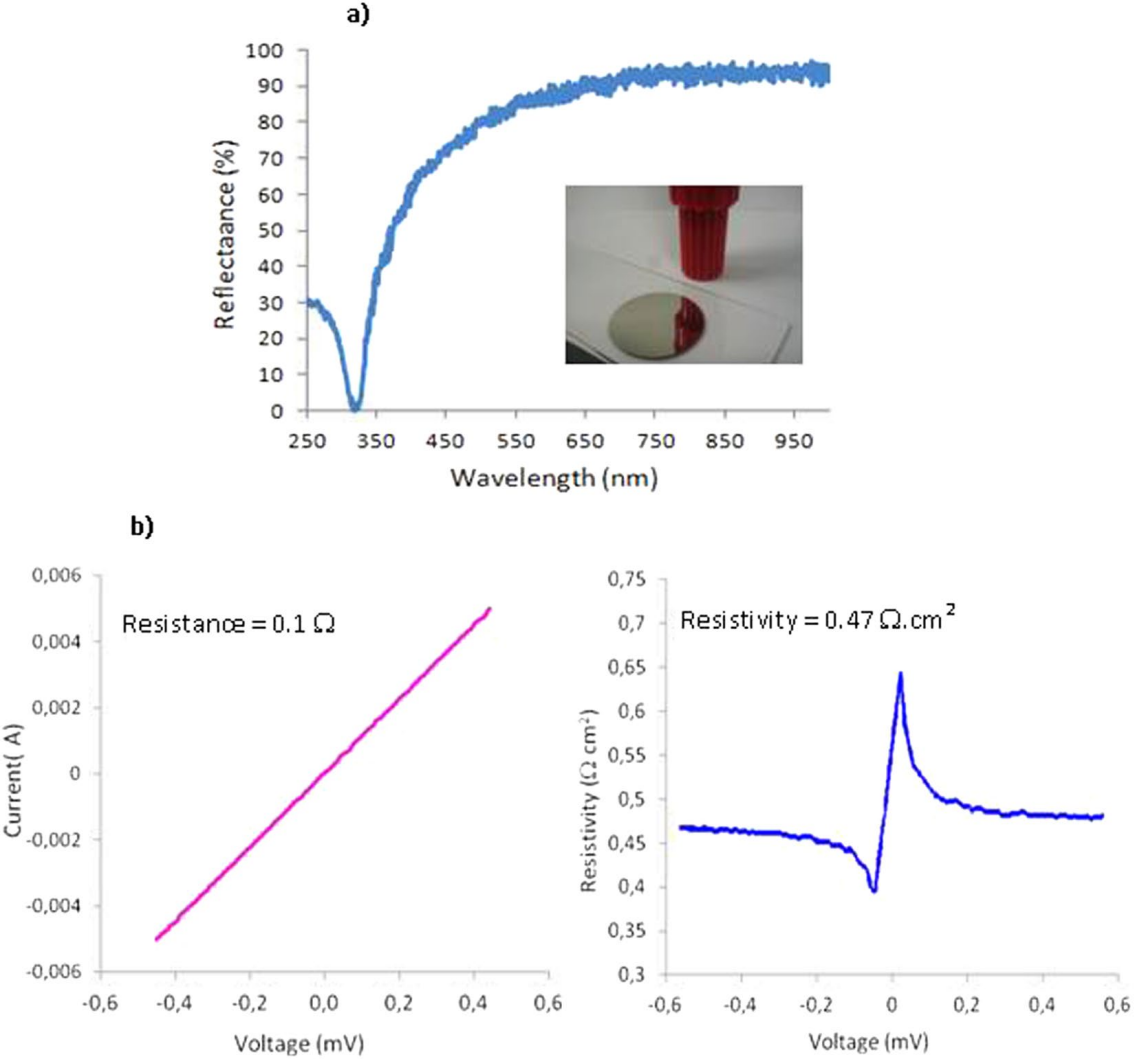
(**a**) Reflectance spectrum of the silver silver@polymer coating (insert: photographic image of the sample). (**b**) Resistive characteristics of the as-developed silver@polymer nanomaterial. Current/tension characteristic (left) and surface resistivity (right) measured by the 4-point technique with a 5 mm distance between the measurement electrodes.

The as-coated silver@polymer nanoassemblies exhibit a pretty smooth surface with evident mirror properties (Fig. 5a). The reflection spectrum of the silver@polymer coating was measured in the 250 to 950 nm spectral range (Fig. 5a); it shows a remarkably high reflectance exceeding 90% in the red, very close to that of optically polished bulk silver^43^. Due to surface plasmon resonance, the reflectance decreases dramatically below 350 nm (Fig. 5a).

As shown by the SEM image (Fig. 3), the surface topography of the layer is fairly regular, with silver nanoparticles sizes lower than 70 nm, uniformly distributed and tightly arranged, without defects or gaps. Moreover, the cross-sectional TEM micrographs reveal a rugosity of the films not exceeding a few nanometers (see Fig. 4). These observations are quite consistent with the macroscopic optical appearance of the layers. This confirms the excellent surface quality of this nanomaterial.

One of the key questions arises as to whether the electric properties of the photo-generated silver@polymer layers can compete with that of currently available concurrent materials and most particularly, their conductivity. Electric characterizations were performed on as-developed coatings synthesized in opened to air conditions with 5 wt% Ag and were evaluated by using the four-point probe technique^44^. As expected, the conductance was observed to strongly increase with increasing the time exposure (from 3 to 30 min). In all likelihood, this behavior is to be related to a better percolation of the AgNPs and to the formation of a quite compact metal film as evidenced by TEM analysis (see Fig. 4e).

The surface resistivity of a sample (30 min exposure) was measured to be 0.47 Ω.cm^2^. By introducing the geometrical characteristics of the 4-point device and a thickness of the silver top layer of ca 250 µm, one can calculate a linear conductivity of 8.6 10^4^ S.cm^−1^ (Fig. 5b). In consideration of the fact that the conductive layer is of submicrometric thickness, consisting of assembled nanosized particles, and with traces of the binder polymer material, such an order of magnitude (only 7 times lower than the standard value of bulk silver 6.6 × 10^5^ S.cm^−1^)^6^, is a great result. Moreover, this excellent conductivity remained constant, even when high intensities were used (20 mA) and the samples did not exhibit any anisotropy of their electric properties.

**Figure 6:**
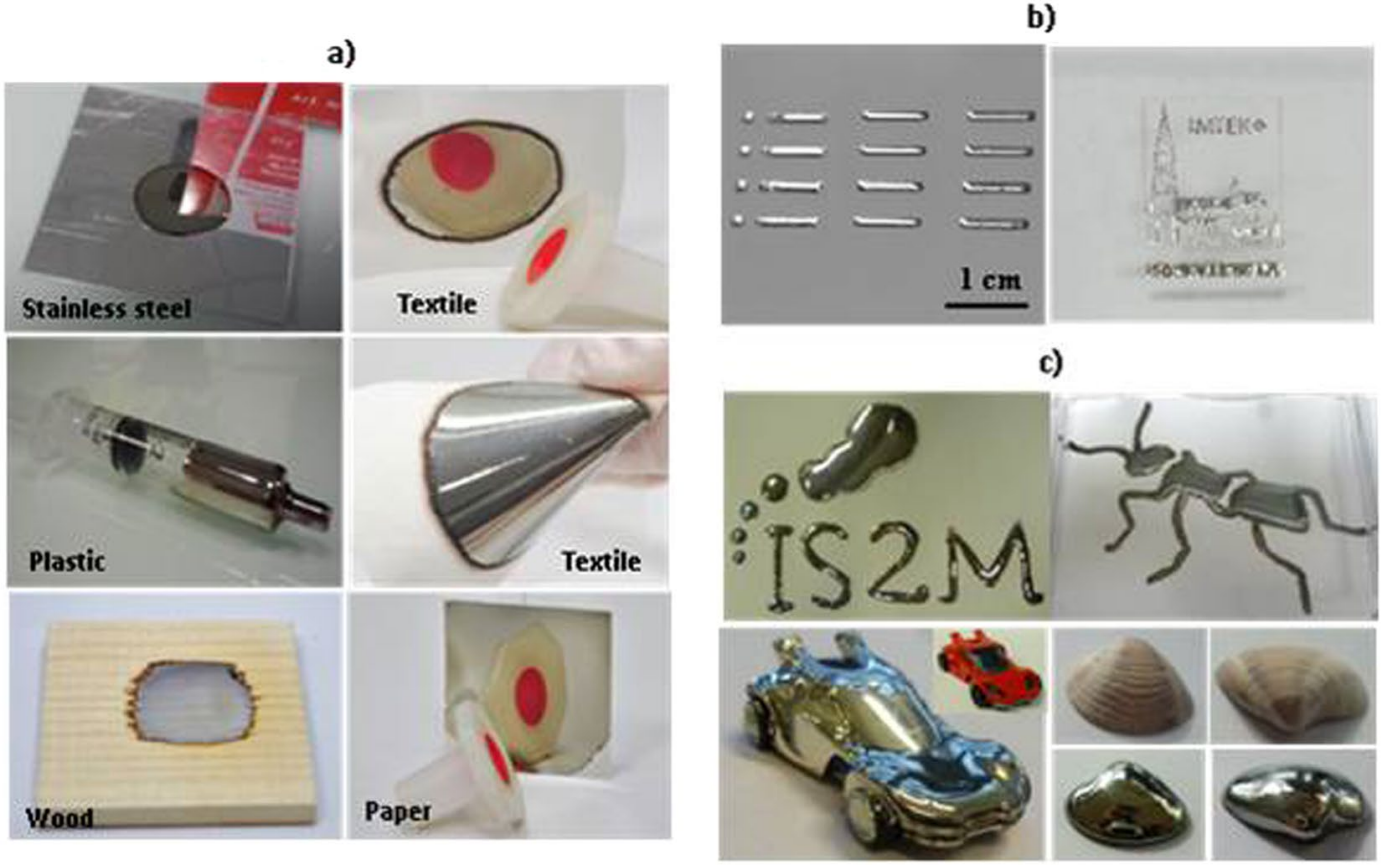
Photographic images of the silver@polymer nanomaterial (**a**) coated on various surfaces, (**b**) printed sample with a digital Dimatix© printer on glass support and (**c**) 2D and 3D coated objects with the silver@polymer nanoassembly.

In order to screen its possible fields of application, the formulation was deposited on a variety of substrates (textile, paper, glass, wood, plastic, aluminum and stainless steel) and exposed to UV for 3 minutes. The deposition of the photosensitive formulation by different methods on the selected surfaces was also reviewed: wire bar coater deposition for the samples on textile, wood and metal surfaces, dip coating for plastic and paper or simple drop deposition and inkjet printing on glass. Silver coatings with high reflectivity were readily obtained on the selected surfaces (Fig. 6a). Moreover, on flexible surfaces such as textile, the flexibility was preserved after coating with silver@polymer nanoassembly (see Fig. 6a) due to the architecture of the nanomaterial (concentration gradient of the AgNPs in polymer). The silver coatings are very stable for at least two years in the absence of any kind of protective surface treatment e.g. transparent varnish. Quite interestingly, the photosensitive formulation can be used as an ink in a numeric inkjet printing device (Fig. 6b **)**. In that way, it was easy to print patterns or images onto various surfaces.

## Conclusion

In conclusion, playing with the light makes it possible not only to generate simultaneously metal nanoparticles and the 3D polymer host but also offers the possibility of introducing a specific spatial organization of the particles in the polymer. The original assembling process highlighted in this paper allows multifunctional reflective and conductive silver@polymer nanomaterials to be produced in a very simple, fast and cheap way that makes them attractive candidates for further developments, both from practical and economical viewpoints.

It is worthy of notice that, owing to its simplicity of use, rapidity of operation and high stability of photogenerated silver@polymer coatings, this innovative approach offers great advantages over concurrent metallization processes by being adaptable to a great variety of substrates e.g. textile, paper, glass, wood, plastic, etc. Moreover, the flexibility of the surfaces as textile or paper, was preserved after coating due to the concentration gradient of AgNPs increasing from the bottom to the top in the polymer. The specific assembling was clearly demonstrated by TEM and XPS analysis.

On a short term, this assembling process should open up new vistas in the field of micro-electronic components, connectors for automobile industries, electrical construction, computer and telephone. The fair optical properties of these coatings in terms of reflectivity and mirror aspect, besides consuming much less silver (only 3 to 5 wt.%) than their concurrent, should also arouse interest for applications in light condensors, solar energy concentrators or adaptative optical devices but also in aesthetic and decorative applications. Moreover, the possibility to apply the as-developed formulation in inkjet printers, coupled to a suitable light source to print reflective and/or conductive patterns adds further to the interest of this innovative multifunctional nanomaterial.

And finally, this photochemical approach should be extended to the design of other metals coatings like gold, palladium, platinum, etc.

## Methods

### Materials synthesis

Silver nitrate (AgNO_3_) with a purity >99% and diphenyl (2,4,6-Trimethylbenzoyl) phosphine oxide were purchased from Sigma-Aldrich and used as received. Polyethylene glycol (600) diacrylate monomer (PEG600DA) was purchased from Sartomer.

**Figure 7:**
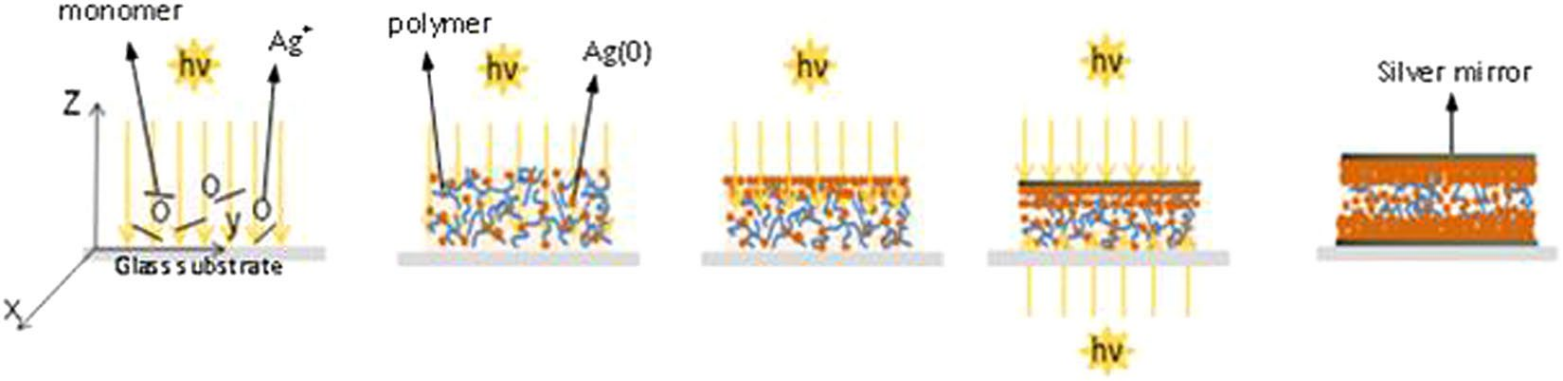
Schematic representation of the photo-induced synthesis and organisation of silver@polymer nanoassemblie.

The photosensible formulation - metal precursor Ag^+^ (5 wt.%), photosensitizer (0.5 wt.%) and acrylate monomer - was leaved for 60 min at ambient temperature under magnetic stirring. This step ensured total dissolution of the metal precursor and photosensitive generator of free radicals. A few drops of this mixture were spread on a horizontal substrate and exposed at 400 mW/cm^2^ for a few minutes. Figure 7 shows the schematic representation of the photo-induced synthesis and organisation of silver@polymer nanoassemblie. In some cases, another synthetic procedure was used; it involved (i) lamination of the sensitive formulation between two glass slides, (ii) illumination of the sample for a few seconds, (iii) removal of the cover slide and (iv) continuation of the illumination in open air as before.

### Material characterization

The photochemical reactions were carried out with a Hamamatsu Lightningcure LC8 (Hg-Xe L8252) device fitted with a 365 nm-elliptical reflector. The experimental set up used to shape up the actinic beam delivered a maximum fluence of 500 mW/cm² in the 300–450 nm range.

UV-Vis spectroscopy was performed in order to visualize the advancement of the synthesis of silver nanoparticles in the polymerizable matrix as a function of the light exposure. The progress of the reaction was monitored via UV-visible absorption spectroscopy with a Thermo Fisher Scientific, Evolution 200 spectrophotometer. An integrating sphere was also used to measure the reflectance spectra from the Thermo Fisher Scientific. Transmission electron microscopy (TEM) was used to characterize the morphology and the assembling of the nanoparticles in the polymer matrix. TEM measurements were carried out at 200 kV using a Philips CM200 instrument with LaB6 cathode. The scanning electron microscopy (SEM) investigations were performed on a FEI Quanta 400 Scanning electron microscope. X-ray photoelectron spectroscopy (XPS) analysis was carried out under ultra-high vacuum (P < 10^−9^ mbar) on a VG Scienta SES 200-2 spectrometer equipped with a monochromatic Al-Ka X-ray source (hv = 1468.6 eV). The measurements were performed at normal incidence (the sample plane is perpendicular to the emission angle). The depth analyzed extends up to about 8 nm. The high resolution spectra and wide scan were recorded with pass energy of 100 eV and 500 eV respectively. The peaks were fitted using CASA-XPS software, after substraction of Shirley-type background. The areas of each component were determinated taking into account the transmission factor of spectrometer, mean free path, and sensibility factor of each atom.

## Electronic supplementary material

Supplementary Information
