# Supplementary material for: Generating highly reflective and conductive metal layers through a light-assisted synthesis and assembling of silver nanoparticles in a polymer matrix
Source: Sci Rep. 2017 Sep 29;7:12410. doi: 10.1038/s41598-017-12617-8 (PMC5622138; doi:10.1038/s41598-017-12617-8)
Supplement: Supplementary file 1 — Supplementary Information [file 41598_2017_12617_MOESM1_ESM.doc]

**Generating highly reflective and conductive metal layers through a light-assisted synthesis and assembling of silver nanoparticles in a polymer matrix**

**Mohamed Zaier, Loïc Vidal, Samar Hajjar-Garreau, Lavinia Balan***

*CNRS, Institut de Science des Matériaux de Mulhouse, UMR 7361, 15 rue Jean Starcky, 68057 Mulhouse, France*

**Corresponding author: E-mail address: lavinia.balan@uha.fr; Tel: +33(0)3 8960 8844; Fax: 33 +33(0) 38960 8799*

**Photo-induced synthesis and organisation of silver@polymer nanoassemblies.**


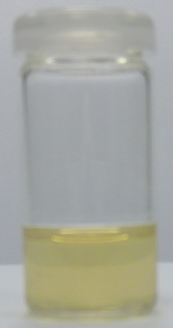


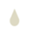

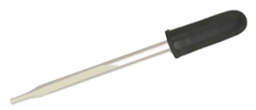


**Figure S1.** Schematic illustration of silver@polymer nanoassembling coating

**Figure S2.** Photographic images of the samples before irradiation and after irradiation on both sides (air and glass sides).


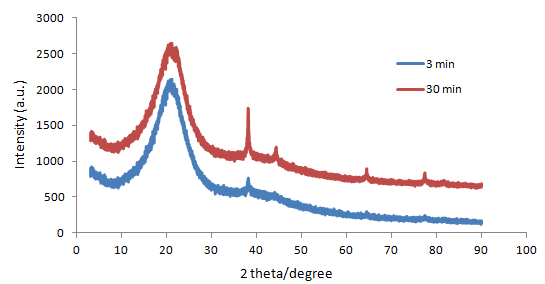


**a)**

**b)**

**Figure S3.** XRD patterns of as-developed nano-assembly **a)** after 3 min UV exposure and **b)** after 30 min UV exposure.

**
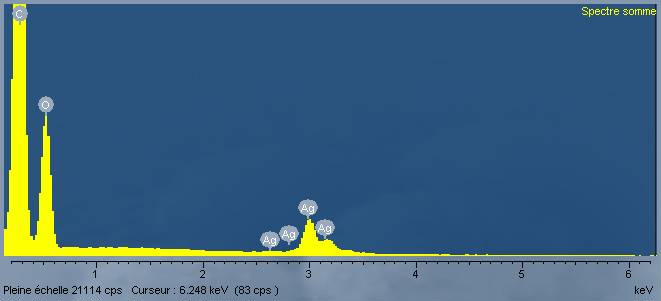
**

**Figure S4:** EDXS spectrum of the nanomaterial.

**Figure S5**. Representative wide-scan XPS spectrum taken from **a)** the surface of the nano-assembly coating film, **b)** in depth of the nano-assembly and also the spectra for the Ag 3d **c)** and d) at the surface and in depth of the nanomaterial respectively.


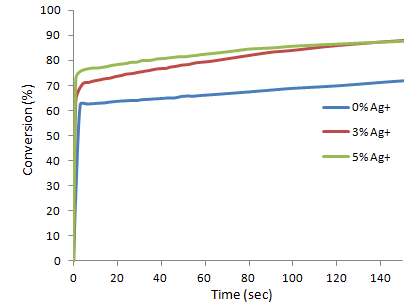


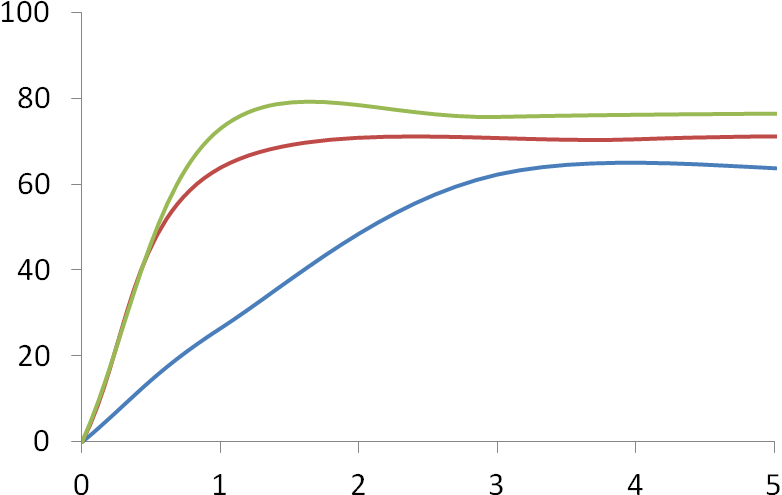


**Figure S6.** Conversion vs time kinetic curves of acrylic formulation with and without Ag+ upon irradiation at ambient temperature in aerated conditions. Thickness = 24 µm, light intensity = 20 mW/cm^2^.
